# Experimental Evidence for the Effect of Small Wind Turbine Proximity and Operation on Bird and Bat Activity

**DOI:** 10.1371/journal.pone.0041177

**Published:** 2012-07-30

**Authors:** Jeroen Minderman, Chris J. Pendlebury, James W. Pearce-Higgins, Kirsty J. Park

**Affiliations:** 1 Biological and Environmental Sciences, University of Stirling, Stirling, United Kingdom; 2 British Trust for Ornithology, The Nunnery, Thetford, United Kingdom; University of Western Ontario, Canada

## Abstract

The development of renewable energy technologies such as wind turbines forms a vital part of strategies to reduce greenhouse gas emissions worldwide. Although large wind farms generate the majority of wind energy, the small wind turbine (SWT, units generating <50 kW) sector is growing rapidly. In spite of evidence of effects of large wind farms on birds and bats, effects of SWTs on wildlife have not been studied and are likely to be different due to their potential siting in a wider range of habitats. We present the first study to quantify the effects of SWTs on birds and bats. Using a field experiment, we show that bird activity is similar in two distance bands surrounding a sample of SWTs (between 6–18 m hub height) and is not affected by SWT operation at the fine scale studied. At shorter distances from operating turbines (0–5 m), bat activity (measured as the probability of a bat “pass” per hour) decreases from 84% (71–91%) to 28% (11–54%) as wind speed increases from 0 to 14 m/s. This effect is weaker at greater distances (20–25 m) from operating turbines (activity decreases from 80% (65–89%) to 59% (32–81%)), and absent when they are braked. We conclude that bats avoid operating SWTs but that this effect diminishes within 20 m. Such displacement effects may have important consequences especially in landscapes where suitable habitat is limiting. Planning guidance for SWTs is currently lacking. Based on our results we recommend that they are sited at least 20 m away from potentially valuable bat habitat.

## Introduction

Increasing awareness of global climate change has led to the rapid proliferation of government targets worldwide to reduce carbon emissions (e.g. in the Kyoto protocol). The generation of power from low-carbon sources is a key element in strategies to meet such targets. As a result, the renewable energy sector has grown rapidly over the last few decades, with wind power forming a major component of this increase. Over 197 GW of power are now generated by wind turbines worldwide [1].

The size and scale of wind turbines varies enormously. The focus of wind energy development has been on wind farms containing multiple large turbines with rotor diameters up to and over 100 m, each generating up to 2.3 MW. However, a more recent development is the expansion of the small wind sector. In the UK, small wind turbines (SWTs) are legally defined as units that can generate up to 50 kW [2]. Usually SWTs are installed singly, and dimensions can vary but the majority of units installed in the UK range from 6 to 12 m hub height [3]. Rapid technological advances have made SWTs increasingly affordable to private land- and homeowners. As a consequence, the number of installed SWTs has grown rapidly over the past decade, with over 16,000 units installed between 2005–2010 in the UK alone, generating over 40 MW [3]. In the US, total installed SWT capacity reached 170 MW [4] in 2010, with global capacity totalling over 440 MW [5].

In spite of well-documented effects of large wind turbines on wildlife, especially birds and bats, to date no studies have investigated similar effects of SWTs. Large wind turbines can cause mortality of birds and bats due to collisions with the tower and moving blades [6], [7], but estimates hereof vary widely between sites [6], [8], [9]. A less well-studied but important further effect is the disturbance or displacement of animals by wind turbines. For example, bird flight lines and activity can be affected by large turbine presence [10]–[12]. Moreover, bird breeding densities [13], [14] and foraging behaviour [15] can be negatively affected by turbine proximity, although other studies show that such effects can not necessarily be generalised across sites [16], [17]. Where they occur, displacement effects can amount to effective habitat loss [6] and may have important consequences on their own. Moreover, an understanding of displacement effects and concurrent behavioural avoidance of turbines is vital in predicting the likely risk of collision [18]. Although there is mounting anecdotal evidence of mortality of birds and bats associated with SWTs [19], to date no studies have investigated their effects on bird and bat flight activity or any resulting displacement. In contrast to large wind turbines, SWTs are often sited near habitat features (e.g. buildings, hedgerows, tree lines) that may be associated with relatively high densities of both birds [20], [21], and bats [22]. Thus, studies of flight activity of birds and bats around SWTs and potential displacement effects are urgently required in order to inform evidence-based planning guidelines for SWT developments.

Here we present the results of the first study to quantify the effects of SWTs on bird and bat flight activity on a local scale (0–25 m). While controlled experiments on large wind turbines are rare and logistically challenging [23]–[25], SWTs offer better opportunities for such tests. We used an experimental approach in which we manipulated SWT operation to test separately the effect of both their proximity and operation on bird and bat flight activity. Specifically, while accounting for possible confounding effects, we test whether:

1. SWT proximity affects bird or bat activity, by comparing activity levels in two distance bands to the turbines, while accounting for the effect of confounding variables (**Test 1**);
2. SWT operation affects bird or bat activity, by comparing activity levels when turbines are operating normally to when they are stopped, while explicitly accounting for the effect of wind speed (**Test 2**); and whether
3. any effect of SWT operation (Test 2) depends on the proximity to the turbine **(Test 3**).

## Materials and Methods

### Data Collection and Experimental Protocol

**Figure 1:**
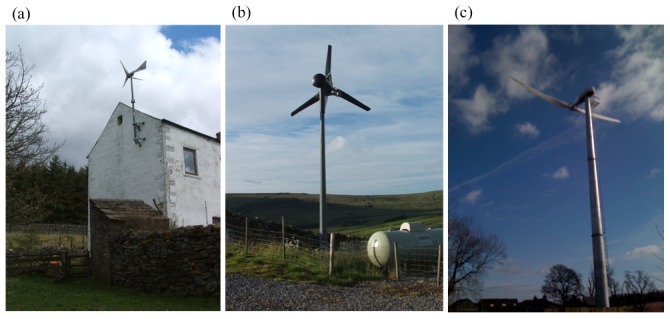
Three examples of turbine models studied. (a) a 10 m high building-mounted model with three blades and a diameter of 1.75 m, (b) a 6.5 m high free-standing model with three blades and a diameter of 3.5 m, (c) a 18 m high free-standing model with two blades and a diameter of 13 m.

We selected twenty SWT sites in central Scotland (N  = 7) and northern England (N  = 13) that represented a range of habitats and included both building-mounted (N  = 5) and free-standing turbines (N  = 15) of 6–18 m hub height (mean  = 8.2 m) and 1.5–13 m blade diameter (mean  = 3.4 m). The majority of turbines studied (18 out of 20) were three-bladed models, the remaining two were twin-bladed models. Taken together, turbine sizes and models studied are an appropriate representation of the range of models installed in the UK (see Figure 1 for examples).

All sites were privately owned and access permission was obtained from home- or landowners prior to the start of data collection. No further permissions were required for the field observations. Between 21 May and 10 September 2010, bird and bat activity data were collected at each site over four successive days and nights (limited to three days and nights at two, and to two days and nights at one site due to access restrictions), and data collection was repeated once during the season at three of the twenty sites. Although we recognise that both bird and bat activity will vary through the season, this does not affect the key tests in the present analysis because activity is compared between experimental treatments (turbines running or braked) over a very short (four days) time span at each site.

To test for effects of turbine operation experimentally, owners were asked to brake their turbine for two 24-hour periods during data collection, ideally alternated with 24-hour periods of normal operation. Due to variation in owner compliance, total braked time per site varied by a maximum of six hours. No braking occurred at two sites, and for a single 24-hour period only at one site.

#### Bird activity

Bird activity was recorded at all 20 sites by a single observer (JM) using two-hour vantage point observations (VPs). One morning (between 4∶30 and 10∶30) and one afternoon (between 16∶30–21∶30) VP took place each day of the four-day period at each site, where possible from a parked car. Observation distances varied due to access restrictions: in the majority of cases this was between 20 and 25 m, with the maximum distance approximately 30 m. In all cases, care was taken to ensure that the area around the turbine was visible and natural flight lines and behaviour were not disturbed. To allow direct comparison with the recording distances in the bat data (see below), the numbers of flights were counted in two distance bands surrounding the turbine (“near”  = 0–10 m and “far”  =  >10–20 m from the turbine). The time, number of individuals, and species was recorded for each flight.

#### Bat activity

Bat activity was automatically recorded using two AnaBat SD2 bat detectors (Titley Scientific, Brisbane, Australia) during all nights of the observation period at each site (bat activity was not obtained at two sites due to detector failure). One (“near”) detector was installed 0–5 m and one (“far”) 20–25 m from the turbine (in one site the “far” detector was only 11 m from the turbine but excluding this site from the analysis did not affect results presented here). All detectors were set to sensitivity setting 6 to avoid interference due to turbine noise, with microphones mounted on a pole at approximately 30–50% of the turbine hub height, and set to record sound from vertically upwards using a deflector plate (a 25×25 Perspex plate angled at 45° to the microphone, which was housed in a 15 cm long tube at 30 cm from the centre of the deflector). This approach eliminated potential overlap in detection ranges between the “near” and “far” detectors because (i) the microphones used are directional and most sensitive in a 90° cone in the recording direction; and (ii) the maximum detection distance of the detectors at the settings used is limited (i.e. approximately 13.7 m, see [26]). All recordings were inspected manually in AnalookW (version 3.7 w, 2009), blinded with respect to site or turbine operation. Sequences of two or more echolocation calls separated by at least one second were classified as a bat “pass”. Echolocation calls were identified to species or genus by frequency and shape [27].

#### Habitat and weather data

Linear habitat features such as hedgerows and fence are important to both bats and birds [20]–[22]. We therefore collected three measures of habitat characteristics at each site: the minimum distance (m) of the turbine to (1) buildings; (2) trees or tree lines (either trees or shrub >3 m in height); and (3) linear features (hedgerows, fence lines, dry stone walls, tree lines, terraced buildings separated by <5 m or a combination of these forming an approximately straight line for at least 10 m). These measurements were obtained from 1∶1250 Ordnance Survey maps, and verified by field observations. Weather conditions also affect both bird and bat activity, and we therefore obtained daily measurements of total rainfall (mm), average wind speed (m/s) and minimum temperature (°C) from Met Office MIDAS weather stations nearest to each site (mean distance 13 km, min-max: 3–28 km, inter-quartile range: 7–20 km) [28]. Because of the key importance of wind speed in the analysis presented here, we confirmed that measurements from weather stations were appropriate proxies for the conditions at the turbine sites by correlating wind speed estimated on the Beaufort scale during a sub-sample of the VPs (N  = 85) to the corresponding wind speed measured at the nearest weather station (Pearson correlation, r  = 0.525, df  = 84, t  = 5.65, p<0.001).

### Statistical Analysis

Bird and bat activity data were analysed using Generalised Linear Mixed Effects Models (GLMMs) [29]. In all models, minimum temperature (°C), rainfall (mm), wind speed (m/s), and distance to buildings, tree lines and linear features were included as fixed covariates, and turbine operation (braked or running) as a fixed factor.

Bird activity (number of flights per hour) was log-transformed to achieve approximate normality of random effects (Shapiro-Wilk test, W  = 0.994, p  = 0.144 in final models), and modelled with normal error distributions. In addition to the above factors, date and date^2^ (to account for potential non-linear changes in activity through the season) were included in the models as covariates, and distance band (“near” or “far”) as a factor. Time of day was included as a factor (with two levels: morning or afternoon) because variation in time of day during VPs was limited (see above). Distance band nested within site was included as a random effect.

**Table 1:** Parameter estimates and likelihood ratio tests of the GLMM for the activity (number of flights per hour) of all bird species combined.

		95% CI						
*Fixed effects:*	Estimate	Lower	Upper	ΔAIC	ΔLog Likelihood	χ^2^	χ^2^ df	*p*
Intercept	6.213	−1.289	13.686					
Wind speed (m/s)	0.016	−0.012	0.044	1.52	0.24	0.48	1	0.4904
Rainfall (mm)	−0.009	−0.029	0.011	0.69	0.65	1.31	1	0.2526
Min. temperature (°C)	0.003	−0.025	0.031	1.55	0.23	0.45	1	0.5008
Time of day ^1^	−0.398	−0.513	−0.282	−40.18	21.09	42.18	1	<0.001
Julian date	−0.035	−0.111	0.043	0.43	0.78	1.57	1	0.2106
Julian date (squared)	<0.001	<0.001	<0.001	0.38	0.81	1.62	1	0.2027
Distance to buildings (m)	−0.003	−0.015	0.008	1.95	0.02	0.05	1	0.8258
Distance to trees (m)	−0.005	−0.01	0.001	−0.02	1.01	2.02	1	0.1549
Distance to linear features (m)	−0.025	−0.05	0.001	−1.45	1.72	3.45	1	0.0634
Operation ^2^	0.123	−0.081	0.329	1.20	0.40	0.80	1	0.3705
Distance band ^3^	−0.109	−0.612	0.395	1.67	0.16	0.33	1	0.5659
Wind * Operation ^2^	−0.023	−0.053	0.007	1.67	0.17	0.33	1	0.5634
Wind * Operation ^4^ * Distance band ^3^	0.002	−0.031	0.035	3.21	0.39	0.79	2	0.6750
Wind * Operation ^2^ * Distance band ^3^	−0.008	−0.031	0.014					
***Random effect variances:***								
Distance band/Site	0.564							
Site	0.103							
Residual	0.315							

Reference categories: ^1^ Time of day  =  PM, ^2^ Operation  =  Running, ^3^ Distance band  =  Near, ^4^ Operation  =  Braked.

The 95% confidence interval represents the quantiles of N  = 5000 simulated draws from the estimated parameter distributions. ΔAIC, ΔLog Likelihood, and χ^2^ are likelihood ratio tests of the deletion of each term from the full model (for the 3-way interaction), from the model including two-way interactions only (two-way interaction term) and from the model with main effects only (main effect terms).

Because the distribution of bat passes per hour was highly skewed (no bats were recorded in 58% of hours, mean in remaining hours  = 12) bat activity was modelled as the probability of a bat pass per hour of observation using a logit-link binomial error distribution. In addition to the factors above, time of night, time of night^2^ (to account for potential non-linear changes in activity during the night) were included as covariates and detector distance (“near” or “far”) as a factor. Night nested within site was included as a random effect.

We performed tests 1–3 as outlined above by assessing the effect of focal explanatory variables. First, the effect of detector distance (bats) or distance band (birds) tested whether turbine proximity affects bat or bird activity respectively (**Test 1**). Second, because wind speed affects turbine operation when not stopped, we tested the effect of turbine operation by including an interaction between wind speed and operation (**Test 2**). Third, we tested whether any effect of operation depended on turbine proximity by including an additional three-way interaction between wind speed, operation and detector distance (bats) or distance band (birds) (**Test 3**). To avoid over-parameterisation, and because the focus of the present analysis was to test the effects of turbine proximity and operation, we did not test for further interactions between factors.

We present results as full models including all explanatory variables to avoid bias due to stepwise deletion of non-significant terms [30]. Instead, we draw inferences on the effect of each parameter by a combination of (i) its estimated distribution obtained by N  = 5000 simulations [29] and (ii) a comparison of a models excluding each parameter in turn and its ‘higher-order’ model (i.e. main effects were tested by removing them from a model excluding all interactions, two-way interactions were tested by excluding them from a model excluding three-way interaction, etc.) using Likelihood Ratio Tests (LRTs) [31].

### Software

GLMMs were fitted using the *lme4* [32] package in R version 2.14.0 [33]. In addition to coefficient point estimates we report the 5% and 95% quantiles of N  = 5000 simulation draws from the estimated parameter distributions, obtained using the sim() function in package *arm* [34].

**Table 2:** Parameter estimates and likelihood ratio tests of the GLMM for the activity (probability of observing a bat pass per hour) of all bat species combined.

		95% CI						
*Fixed effects:*	Estimate	Lower	Upper	ΔAIC	ΔLog Likelihood	χ^2^	χ^2^ df	*p*
Intercept	−79.574	−88.381	−70.751					
Wind speed (m/s)	0.103	−0.012	0.218	3.43	−4.86	6.86	1	0.0088
Rainfall (mm)	0.008	−0.045	0.064	<0.01	2.00	0.00	1	0.9632
Min. temperature (°C)	0.011	−0.05	0.072	0.12	1.76	0.24	1	0.6242
Time of night	6.577	5.851	7.309	0.85	0.30	1.70	1	0.1929
Time of night (squared)	−0.135	−0.149	−0.120	220.47	−438.95	440.95	1	<0.0001
Distance to building (m)	−0.011	−0.021	−0.001	2.02	−2.04	4.04	1	0.0445
Distance to trees (m)	−0.011	−0.015	−0.006	3.94	−5.89	7.89	1	0.0050
Distance to linear features (m)	−0.008	−0.027	0.011	0.10	1.80	0.20	1	0.6555
Operation^1^	0.842	0.309	1.376	0.44	1.12	0.88	1	0.3469
Detector^2^	0.250	−0.100	0.601	0.29	1.43	0.57	1	0.4496
Wind * Operation^1^	−0.176	−0.306	−0.050	5.47	−8.95	10.95	1	0.0009
Wind * Operation^3^ * Detector^2^	−0.050	−0.148	0.047	4.38	−4.76	8.76	2	0.0125
Wind * Operation^1^ * Detector^2^	−0.112	−0.185	−0.035					
***Random effect variances:***								
Night within Site	0.897							
Site	<0.001							
Residual	1.000							

Reference categories: ^1^ Operation  =  Running, ^2^ Detector  =  Near, ^3^ Operation  =  Braked.

The 95% confidence interval represents the quantiles of N  = 5000 simulated draws from the estimated parameter distributions. ΔAIC, ΔLog Likelihood, and χ^2^ are likelihood ratio tests of the deletion of each term from the full model (for the 3-way interaction), from the model including 2-way interactions only (2-way interaction term) and from the model with main effects only.

**Figure 2:**
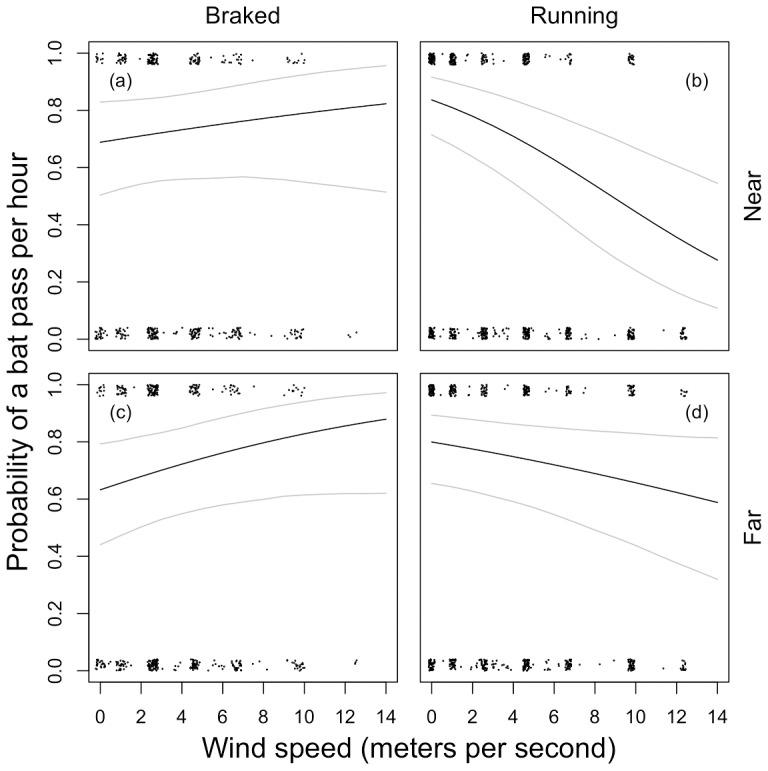
Activity of all bat species combined (probability of observing a bat pass) plotted against wind speed at the “near” (a & b, 0–5 m) and “far” (c & d, 20–25 m) bat detectors, for when turbines are braked (a & c) and running (b & d). Dots are observed data (jittered for better visibility). Black lines are the predicted probabilities of a bat pass from the model in Table 2 and grey lines are the upper and lower 95% prediction intervals obtained from N  = 5000 simulated draws from the estimated parameter distributions. The predictions are made at the median observed values for other parameters in the model.

## Results

### Bird Activity

Across all 20 sites, N  = 12,109 flights (16.3% *Corvidae* spp., 72.1% other passerines, 11.6% non-passerines) were recorded during a total of 354 hours (178 VPs). Between four and 16 VPs were obtained per site.

With the exception of time of day, none of the factors tested had a significant effect on bird activity (Table 1). Thus, accounting for confounding effects, there was no evidence that bird activity was affected by turbine proximity (no effect of distance band; **Test 1**), turbine operation (**Test 2**) or that the effect of turbine operation differed between the two distance bands (**Test 3**). These findings were not affected by analysing activity of species groups separately (Table S1).

### Bat Activity

Across all 18 sites, N  = 8221 bat passes (87.6% *Pipistrellus* spp. 12.4% *Myotis* spp., *Nyctalus noctula* Schreber or *Plecotus auritus* Linnaeus) were recorded in a total of 703 hours (67 nights). Between 19 and 244 hours were sampled per site, during which time turbines were braked between 6 and 102 hours.

The inclusion of wind speed, time of night^2^, distance to building and distance to trees all significantly improved the fit of models for bat activity. While we found no independent effects of either SWT proximity (no effect of detector distance; **Test 1**), the model was significantly improved by an effect of operation dependent on wind speed (significant effect of two-way interaction, **Test 2**), which in turn was modulated by turbine proximity (significant effect of three-way interaction, **Test 3**; Table 2). These findings were similar when analysed separately for *Pipistrellus* spp. and other bat species (Table S2). In close proximity to the turbines, as wind speed increases from 0 to 14 m/s (0–5 m, “near” detectors), this results in a non-significant increase in the probability of a bat pass from 69% (50–83%) to 82% (49–96%) when the turbines are braked (Figure 2a). By contrast, when turbines are running, the same increase in wind speed results in a significant *decrease* in the probability of a bat pass from 84% (71–91%) to 28% (11–54%) (Figure 2b). Although the predicted effect is similar at greater distances from the turbines (20–25 m, “far” detectors) when they are braked (Figure 2c), when they are running the negative effect of wind speed on the probability of a bat pass is much weaker; from 80% (65–89%) to 59% (32–81%) as wind speed increases from 0 to 14 m/s (Figure 2d).

## Discussion

We here present the results of the first study to investigate the impact of small wind turbines on wildlife. Using a field experiment, we tested whether SWT proximity and operation affects the activity of birds and bats in the immediate vicinity of the operating- and non-operating turbines.

### Bird Activity

Accounting for a range of possibly confounding effects and variation among sites, we found that bird activity (the number of flights per hour) was similar in two distance bands surrounding SWTs (**Test 1**). Moreover, bird activity was unaffected by experimental changes in turbine operation while explicitly accounting for variation in wind speed (**Test 2**). Finally, we found no evidence that any effect of turbine operation was modulated by distance or proximity to the turbine (**Test 3**). These findings were similar for all species groups and therefore suggest that neither SWT proximity nor operation significantly affects bird activity at the fine scale studied. Studies of large wind turbines report varying effects of turbine presence or proximity on bird flight behaviour [12], [35], [36], suggesting a high degree of variability among sites and species [37]. Fewer studies have specifically examined the effect of turbine operation on bird activity, and seem to suggest mixed effects, even on the same species [10], [11], [23]. Our results suggest that birds do not avoid the immediate area (within 20 m) around SWTs, implying that turbine presence does not affect habitat use. Although the lack of fine-scale avoidance may mean birds are more susceptible to collisions [18], the level of mortality through collisions with SWTs has yet to be quantified. Alternatively, it is possible that displacement does occur, but at a different spatial scale than studied here.

### Bat Activity

While accounting for confounding effects of weather and habitat, we found that although there was no overall effect of SWT proximity (**Test 1**), bat activity is lower when turbines are running (**Test 2**) and this effect depends on SWT proximity (**Test 3**). These findings were similar when *Pipistrellus* spp. and other species were analysed separately. Bat activity decreases with increasing wind speed when the turbines are running, but not when they are braked. While this decrease is predicted to be substantial (on average 56% over a 14 m/s wind speed increase) at short distances to the turbine (0–5 m), it diminishes at longer distances (20–25 m) from the turbines (on average 21%). This is the first study to quantify variation in bat activity near SWTs, and these findings suggest that areas in their immediate vicinity are selectively avoided by bats, especially when operating and at higher wind speeds.

Studies of large turbines have not shown significant avoidance by bats. Indeed, bats will forage near to (and actively investigate) operating turbine blades [38]. By contrast, we suggest bat activity may be lowered by SWT operation for a variety of reasons. Firstly, there is experimental evidence that the reflection of echolocation pulses off spinning SWT blades can be erratic [39], therefore affecting detection and possibly causing echolocating bats to avoid poorly detected (and thus potentially risky) objects. Alternatively, foraging behaviour and feeding success of some bat species may be affected by ambient noise [40]. Although there is no evidence that large turbines emit significant levels of (ultrasonic) noise [41], it has yet to be tested whether this is the case for SWTs and noise may therefore negatively affect bat activity in their vicinity.

Lower bat activity in the vicinity of operating SWTs implies reduced usage of the habitat surrounding the turbines. While such curtailment of habitat use may have no wider (population-level) effects in less suitable habitats, habitats such as hedgerows and tree lines are known to be important for bats as commuting- and foraging routes [22], [42]. Especially in landscapes where such habitats are rare (e.g. degraded urban or intensive agricultural landscapes) limitations in the opportunity to use them due to the proximity and operation of a SWT may have consequences for foraging success, could lead to the loss of commuting routes, and may therefore affect bat populations in the wider area. The relatively small number of sites studied limits our current ability to test the effect of SWTs in specific habitat contexts. However, it is worth stressing that strong effects of SWT operation on bat activity were only evident in their close proximity, and distance between them and potentially valuable habitat features may therefore mediate any negative effects.

### Conclusions and Implications

In summary, we conclude that although bird activity is not affected by SWT proximity or operation at the fine scale studied, at higher wind speed bat activity decreases in close proximity to operating SWTs. It should be stressed that although we have not investigated the effect of turbine size or model [9], [43], such variation would not alter our conclusions regarding SWT operation because we have studied its effect in a paired experimental design. Similarly, although we found no effects of SWT proximity on bird activity, further studies are necessary to quantify their effects on bird breeding densities or foraging behaviour, both of which can be affected by large turbines [13]–[15].

These caveats aside, the findings presented here have important implications for planning decisions regarding SWTs. Presently, siting guidance for SWTs is extremely limited, both in the UK and elsewhere. For example, in the UK, the limited guidance that does exist suggests siting SWTs away from protected areas (e.g. SSSI’s, SAC’s or SPA’s), away from roost sites, or not within a minimum distance of features that could be used as nest-, roost- or foraging sites [19], [44]. To date, data to support such guidelines have been lacking. We provide the first evidence to show that bat activity is reduced in the immediate vicinity (0–5 m) but not at longer distances (20–25 m) from operating SWTs, suggesting that they can affect habitat use by bats. It may be argued that the wider consequences of the loss of a relatively small area of habitat surrounding operating SWTs would be limited. However, we suggest that especially in landscapes with little suitable habitat, any effects of SWTs that cause the displacement of bats away from the few available commuting routes or foraging areas could have wider population-level impacts. While further work to identify such effects in specific habitat contexts is necessary, we support planning guidelines that recommend siting SWTs at least 20 m away from potentially suitable bat habitat, especially in more degraded landscapes.

## Supporting Information

Table S1 Parameter estimates and likelihood ratio tests of the GLMMs for the activity (number of flights per hour) of (a) *Corvidae*, (b) other passerines and (c) other species.
(DOC)

Table S2 Parameter estimates and likelihood ratio tests of the GLMMs for the probability of a bat pass per hour, of (a) *Pipistrellus* spp. only, and (b) other species only.
(DOC)
