# Supplementary material for: Experimental Evidence for the Effect of Small Wind Turbine Proximity and Operation on Bird and Bat Activity
Source: PLoS One. 2012 Jul 30;7(7):e41177. doi: 10.1371/journal.pone.0041177 (PMC3408485; doi:10.1371/journal.pone.0041177)
Supplement: Table S1 — Parameter estimates and likelihood ratio tests of the GLMMs for the activity (number of flights per hour) of (a) Corvidae, (b) other passerines and (c) other species. (DOC) [file pone.0041177.s001.doc]

**Table S1.** Parameter estimates and likelihood ratio tests of the GLMMs for the activity (number of flights per hour) of (a) *Corvidae*, (b) other passerines and (c) other species. The 95% confidence interval represents the quantiles of N = 5000 simulated draws from the estimated parameter distributions. ΔAIC, ΔLog Likelihood, and χ^2^ are likelihood ratio tests of the deletion of each term from the full model.

| **Table S1(a, *Corvidae*)** |  | **95% CI** | |  |  |  |  |  |
| --- | --- | --- | --- | --- | --- | --- | --- | --- |
| ***Fixed effects:*** | **Estimate** | **Lower** | **Upper** | **ΔAIC** | **ΔLog Likelihood** | **χ^2^** | **χ^2^ df** | ***p*** |
| Intercept | -0.826 | -8.637 | 7.127 |  |  |  |  |  |
| Wind speed (m/s) | 0.002 | -0.025 | 0.03 | -1.31 | 1.66 | 3.31 | 1 | 0.0688 |
| Rainfall (mm) | 0.013 | -0.008 | 0.033 | 0.13 | 0.93 | 1.87 | 1 | 0.1717 |
| Min. temperature (°C) | -0.013 | -0.04 | 0.015 | 0.99 | 0.51 | 1.01 | 1 | 0.3146 |
| Time of day ^1^ | -0.354 | -0.469 | -0.242 | -33.12 | 17.56 | 35.12 | 1 | < 0.001 |
| Julian date | 0.023 | -0.06 | 0.105 | 1.59 | 0.21 | 0.41 | 1 | 0.5217 |
| Julian date (squared) | < 0.001 | < 0.001 | < 0.001 | 2.09 | -0.04 | 0 | 1 | > 0.999 |
| Distance to building (m) | -0.007 | -0.022 | 0.008 | 1.38 | 0.31 | 0.62 | 1 | 0.4301 |
| Distance to trees (m) | -0.003 | -0.01 | 0.004 | 1.23 | 0.38 | 0.77 | 1 | 0.3812 |
| Distance to linear features (m) | -0.013 | -0.046 | 0.019 | 1.42 | 0.29 | 0.58 | 1 | 0.4469 |
| Operation ^2^ | -0.032 | -0.225 | 0.171 | 1.81 | 0.09 | 0.19 | 1 | 0.6657 |
| Distance band ^3^ | -0.037 | -0.351 | 0.286 | 1.96 | 0.02 | 0.04 | 1 | 0.8432 |
| Wind * Operation ^2^ | 0.01 | -0.02 | 0.039 | 1.96 | 0.02 | 0.04 | 1 | 0.8374 |
| Wind * Operation ^4^ * Distance band ^3^ | 0.003 | -0.029 | 0.034 | 3.94 | 0.03 | 0.06 | 2 | 0.97 |
| Wind * Operation ^2^ * Distance band ^3^ | 0.001 | -0.019 | 0.023 |
| ***Random effect variances:*** |  |  |  |  |  |  |  |  |
| Distance band / Site | 0.154 |  |  |  |  |  |  |  |
| Site | 0.555 |  |  |  |  |  |  |  |
| Residual | 0.302 |  |  |  |  |  |  |  |

| **Table S1 (b, other passerines)** |  | **95% CI** | |  |  |  |  |  |
| --- | --- | --- | --- | --- | --- | --- | --- | --- |
| ***Fixed effects:*** | **Estimate** | **Lower** | **Upper** | **ΔAIC** | **ΔLog Likelihood** | **χ^2^** | **χ^2^ df** | ***p*** |
| Intercept | 6.028 | -1.632 | 13.346 |  |  |  |  |  |
| Wind speed (m/s) | 0.02 | -0.011 | 0.05 | -1.23 | 1.62 | 3.23 | 1 | 0.0722 |
| Rainfall (mm) | -0.013 | -0.035 | 0.01 | -0.11 | 1.06 | 2.11 | 1 | 0.1463 |
| Min. temperature (°C) | -0.002 | -0.032 | 0.028 | 1.88 | 0.06 | 0.12 | 1 | 0.724 |
| Time of day ^1^ | -0.371 | -0.496 | -0.243 | -28.36 | 15.18 | 30.36 | 1 | < 0.001 |
| Julian date | -0.037 | -0.113 | 0.043 | -1.15 | 1.57 | 3.15 | 1 | 0.0761 |
| Julian date (squared) | 0 | 0 | 0 | 0.62 | 0.69 | 1.38 | 1 | 0.2408 |
| Distance to building (m) | -0.004 | -0.015 | 0.007 | 1.64 | 0.18 | 0.36 | 1 | 0.5485 |
| Distance to trees (m) | -0.005 | -0.01 | 0.001 | 0.1 | 0.95 | 1.9 | 1 | 0.1684 |
| Distance to linear features (m) | -0.023 | -0.047 | 0 | -1.03 | 1.52 | 3.03 | 1 | 0.0817 |
| Operation ^2^ | 0.097 | -0.132 | 0.32 | -1.74 | 1.87 | 3.74 | 1 | 0.0532 |
| Distance band ^3^ | 0.075 | -0.447 | 0.615 | 1.78 | 0.11 | 0.22 | 1 | 0.6412 |
| Wind * Operation ^2^ | -0.036 | -0.069 | -0.003 | 2.11 | -0.05 | 0 | 1 | > 0.999 |
| Wind * Operation ^4^ * Distance band ^3^ | -0.008 | -0.045 | 0.029 | 3.3 | 0.35 | 0.7 | 2 | 0.7035 |
| Wind * Operation ^2^ * Distance band ^3^ | -0.01 | -0.034 | 0.014 |
| ***Random effect variances:*** |  |  |  |  |  |  |  |  |
| Distance band / Site | 0.705 |  |  |  |  |  |  |  |
| Site | < 0.001 |  |  |  |  |  |  |  |
| Residual | 0.388 |  |  |  |  |  |  |  |
|  |  |  |  |  |  |  |  |  |

| **Table S1 (c, other bird species)** |  | **95% CI** | |  |  |  |  |  |
| --- | --- | --- | --- | --- | --- | --- | --- | --- |
| ***Fixed effects:*** | **Estimate** | **Lower** | **Upper** | **ΔAIC** | **ΔLog Likelihood** | **χ^2^** | **χ^2^ df** | ***p*** |
| Intercept | 4.273 | -1.476 | 10.047 |  |  |  |  |  |
| Wind speed (m/s) | -0.005 | -0.032 | 0.021 | -1.9 | 1.95 | 3.9 | 1 | 0.0483 |
| Rainfall (mm) | -0.001 | -0.021 | 0.018 | 2 | 0 | 0 | 1 | 1 |
| Min. temperature (°C) | 0.012 | -0.014 | 0.038 | 1.44 | 0.28 | 0.56 | 1 | 0.4553 |
| Time of day ^1^ | -0.153 | -0.266 | -0.043 | -5.31 | 3.65 | 7.31 | 1 | 0.0069 |
| Julian date | -0.032 | -0.092 | 0.029 | -4.21 | 3.11 | 6.21 | 1 | 0.0127 |
| Julian date (squared) | 0 | 0 | 0 | 1.08 | 0.46 | 0.92 | 1 | 0.3366 |
| Distance to building (m) | -0.001 | -0.009 | 0.006 | 2.1 | -0.05 | 0 | 1 | 1 |
| Distance to trees (m) | 0 | -0.003 | 0.004 | 2.24 | -0.12 | 0 | 1 | 1 |
| Distance to linear features (m) | -0.005 | -0.022 | 0.011 | 1.63 | 0.18 | 0.37 | 1 | 0.5437 |
| Operation ^2^ | -0.117 | -0.309 | 0.082 | 1.94 | 0.03 | 0.06 | 1 | 0.8128 |
| Distance band ^3^ | -0.239 | -0.522 | 0.046 | -1.04 | 1.52 | 3.04 | 1 | 0.0813 |
| Wind * Operation ^2^ | 0.02 | -0.008 | 0.049 | -1.04 | 1.52 | 3.04 | 1 | 0.0811 |
| Wind * Operation ^4^ * Distance band ^3^ | 0.007 | -0.023 | 0.037 | 3.72 | 0.14 | 0.28 | 2 | 0.8683 |
| Wind * Operation ^2^ * Distance band ^3^ | 0 | -0.021 | 0.022 |
| ***Random effect variances:*** |  |  |  |  |  |  |  |  |
| Distance band / Site | 0.124 |  |  |  |  |  |  |  |
| Site | 0.072 |  |  |  |  |  |  |  |
| Residual | 0.284 |  |  |  |  |  |  |  |
|  |  |  |  |  |  |  |  |  |

Reference categories: ^1^ Time of day = PM, ^2^ Operation = Running, ^3^ Distance band = Near, ^4^ Operation = Braked.
