# Supplementary material for: Experimental Evidence for the Effect of Small Wind Turbine Proximity and Operation on Bird and Bat Activity
Source: PLoS One. 2012 Jul 30;7(7):e41177. doi: 10.1371/journal.pone.0041177 (PMC3408485; doi:10.1371/journal.pone.0041177)
Supplement: Table S2 — Parameter estimates and likelihood ratio tests of the GLMMs for the probability of a bat pass per hour, of (a) Pipistrellus spp. only, and (b) other species only. (DOC) [file pone.0041177.s002.doc]

**Table S2.** Parameter estimates and likelihood ratio tests of the GLMMs for the probability of a bat pass per hour, of (a) *Pipistrellus* spp. only, and (b) other species only. The 95% confidence interval represents the quantiles of N = 5000 simulated draws from the estimated parameter distributions. ΔAIC, ΔLog Likelihood, and χ^2^ are likelihood ratio tests of the deletion of each term from the full model.

| **Table S2(a) *Pipistrellus* spp. only** |  | **95% CI** | |  |  |  |  |  |
| --- | --- | --- | --- | --- | --- | --- | --- | --- |
| ***Fixed effects:*** | **Estimate** | **Lower** | **Upper** | **ΔAIC** | **ΔLog Likelihood** | **χ^2^** | **χ^2^ df** | ***p*** |
| Intercept | -72.483 | -81.15 | -63.936 |  |  |  |  |  |
| Wind speed (m/s) | 0.064 | -0.051 | 0.181 | 3.40 | -4.81 | 6.81 | 1 | 0.0091 |
| Rainfall (mm) | 0.007 | -0.046 | 0.061 | 0.01 | 1.98 | 0.02 | 1 | 0.8840 |
| Min. temperature (°C) | 0.035 | -0.027 | 0.094 | 0.80 | 0.41 | 1.59 | 1 | 0.2073 |
| Time of night | 5.985 | 5.288 | 6.692 | 1.14 | -0.28 | 2.28 | 1 | 0.1312 |
| Time of night (squared) | -0.123 | -0.137 | -0.108 | 187.00 | -372.01 | 374.01 | 1 | < 0.0001 |
| Distance to building (m) | -0.016 | -0.026 | -0.005 | 3.66 | -5.32 | 7.32 | 1 | 0.0068 |
| Distance to trees (m) | -0.01 | -0.015 | -0.005 | 3.62 | -5.23 | 7.23 | 1 | 0.0072 |
| Distance to linear features (m) | -0.005 | -0.024 | 0.014 | < 0.01 | 2.00 | < 0.01 | 1 | 0.9499 |
| Operation^1^ | 0.642 | 0.133 | 1.161 | 0.17 | 1.67 | 0.33 | 1 | 0.5645 |
| Detector^2^ | 0.164 | -0.176 | 0.509 | 0.54 | 0.92 | 1.08 | 1 | 0.2992 |
| Wind * Operation^1^ | -0.129 | -0.258 | -0.002 | 3.33 | -4.66 | 6.66 | 1 | 0.0099 |
| Wind * Operation^3^ * Detector^2^ | -0.032 | -0.133 | 0.067 | 3.59 | -3.18 | 7.18 | 2 | 0.0276 |
| Wind * Operation^1^ * Detector^2^ | -0.101 | -0.177 | -0.027 |  |  |  |  |  |
|  |  |  |  |  |  |  |  |  |
| ***Random effect variances:*** |  |  |  |  |  |  |  |  |
| Night within Site | 0.898 |  |  |  |  |  |  |  |
| Site | < 0.001 |  |  |  |  |  |  |  |
| Residual | 1.000 |  |  |  |  |  |  |  |

Reference categories: ^1^ Operation = Running, ^2^ Detector = Near, ^3^ Operation = Braked.

| **Table S2(b) Other bat spp. only** |  | **95% CI** | |  |  |  |  |  |
| --- | --- | --- | --- | --- | --- | --- | --- | --- |
| ***Fixed effects:*** | **Estimate** | **Lower** | **Upper** | **ΔAIC** | **ΔLog Likelihood** | **χ^2^** | **χ^2^ df** | ***p*** |
| Intercept | -77.111 | -91.441 | -62.835 |  |  |  |  |  |
| Wind speed (m/s) | 0.140 | -0.023 | 0.298 | 1.28 | -0.57 | 2.57 | 1 | 0.1091 |
| Rainfall (mm) | 0.020 | -0.053 | 0.091 | 0.10 | 1.80 | 0.2 | 1 | 0.6509 |
| Min. temperature (°C) | -0.043 | -0.122 | 0.037 | 0.49 | 1.01 | 0.99 | 1 | 0.3199 |
| Time of night | 6.195 | 5.025 | 7.370 | 0.90 | 0.21 | 1.79 | 1 | 0.1808 |
| Time of night (squared) | -0.127 | -0.151 | -0.103 | 78.73 | -155.46 | 157.46 | 1 | < 0.001 |
| Distance to building (m) | 0.003 | -0.017 | 0.023 | 0.00 | 2.00 | < 0.01 | 1 | 0.9890 |
| Distance to trees (m) | -0.016 | -0.029 | -0.004 | 2.50 | -3.00 | 5.00 | 1 | 0.0254 |
| Distance to linear features (m) | 0.001 | -0.035 | 0.037 | < 0.01 | 2.00 | < 0.01 | 1 | 0.9499 |
| Operation^1^ | 0.883 | 0.111 | 1.635 | 1.42 | -0.83 | 2.83 | 1 | 0.0923 |
| Detector^2^ | 0.781 | 0.296 | 1.267 | 0.96 | 0.08 | 1.92 | 1 | 0.1659 |
| Wind * Operation^1^ | -0.169 | -0.338 | 0.006 | 1.41 | -0.83 | 2.83 | 1 | 0.0927 |
| Wind * Operation^3^ * Detector^2^ | -0.180 | -0.320 | -0.038 | 4.11 | -4.23 | 8.23 | 2 | 0.0164 |
| Wind * Operation^1^ * Detector^2^ | -0.121 | -0.220 | -0.021 |  |  |  |  |  |
|  |  |  |  |  |  |  |  |  |
| ***Random effect variances:*** |  |  |  |  |  |  |  |  |
| Night within Site | 0.685 |  |  |  |  |  |  |  |
| Site | 1.125 |  |  |  |  |  |  |  |
| Residual | 1.000 |  |  |  |  |  |  |  |

Reference categories: ^1^ Operation = Running, ^2^ Detector = Near, ^3^ Operation = Braked.
